# Spontaneous Amputation below the Knee

**Published:** 1860-01

**Authors:** John G. Kerr

**Affiliations:** Canton, China


### ART. IV.—*Spontaneous Amputation below the Knee.* By JOHN G. KERR, M.D., of Canton, China.

THE subject of this notice is a Chinese, now forty-three years of age, a native of the District of San Ui, in Canton Province. He was for several years employed by foreigners in Hong Kong and Canton, in the various capacities of coolie, servant, and cook. His constitution was good but not robust, and he had always enjoyed good health. He was active and attentive in doing his work.

About the month of June, 1848, when he was thirty-two years old, his left leg was attacked with an inflammatory swelling, which began just above the knee, and extended until the whole limb became involved. When the disease had continued about three weeks, he was admitted into the Rev. Dr. P. Parker's Hospital, where he was under treatment one month. Not being relieved, and refusing to submit to amputation, he was removed to his native place, about seventy-five miles from Canton. At this time his limb was much enlarged and very painful. He was excessively emaciated, and his appearance was withal so haggard, that those interested in him had no thought of ever seeing him again.

For two months after his removal to the country he was treated by native doctors, and still receiving no benefit, he gave up all professional treatment. About this time an abscess opened just above the knee and a little to the inner side of the thigh. This discharged profusely for a time, but without effectual relief. The inflammatory action in the tissues of the leg had resulted in such changes of structure as to interfere with the circulation and innervation of the limb; and about the middle of November mortification took place. He says this occurred without much change of color, but the skin lost its sensibility, became cold, and then the toes, foot, and flesh dropped off one after another, and in three or four days the bones of the leg were left bare and dry. There was no hemorrhage, and it seems that at the line of separation very little suppuration occurred. The abscess above the knee now gradually healed, and the general health improved. About four months more were required to cast off the bones of the leg; this took place in March, 1849, and in two months more the end of the stump was covered with skin.

The stump is now six inches long below the knee, and three and a third inches in diameter at its middle. The bone separated obliquely, leaving the end shaped somewhat like the point of a pen; and, as there was no muscle to cover it, the end is quite conical and sharp. The new skin over the end of the bone is thin, and shows irregularities of surface at the place of separation, but it has not been abraded since it first healed up. A cicatrice just above the knee marks the place where the abscess was discharged.

The stump is held at right angles with the thigh by the contraction of the flexor muscles, but the knee-joint is not anchylosed. The motion is free, as far as the contracted muscles will admit of it.

The particulars of the above case I have learned, by careful inquiry, from the man himself, and from the gentlemen in whose employ he was at the time of and before his sickness. I have depended upon his memory for the dates, and I believe them to be correct, or as nearly so as could be expected after the lapse of so long a time.

The interest of the case would be increased if a full report of it had been made at the time of its occurrence; but the facts as above stated are sufficient to show how much nature is able to accomplish under the most unfavorable circumstances. It also shows how much suffering men have to endure who have not the aid of scientific medicine and surgery. I see multitudes of cases where very simple operations would have prevented long-continued suffering. Only a few days since I extracted an iron bullet from a man's shoulder which had been there for more than four years. The cases where caries of the bones causes protracted irritation are very numerous, and for such, native physicians have no adequate remedies.

